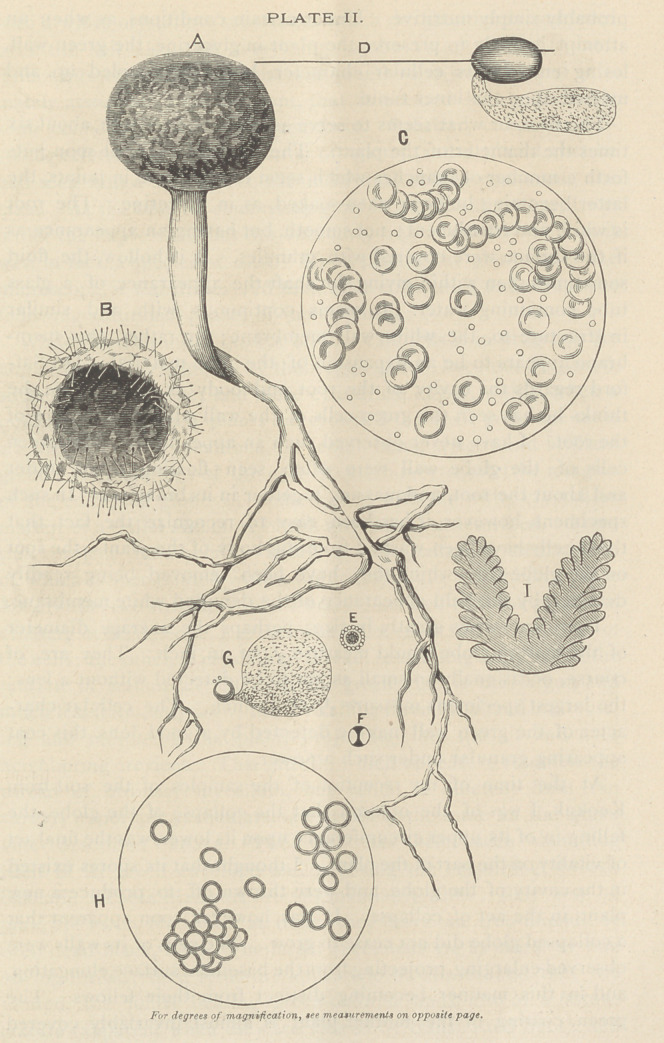# On a Marsh Plant from the Mississippi River Ague Bottoms, Supposed to Be Kindred to the Gemiasma of Salisbury; with a Consideration of Its Genetic Relations to Malarial Diseases

**Published:** 1874-01

**Authors:** John Bartlett

**Affiliations:** Chicago


A MONTHLY RECORD OF

*Medicine, Surgery and the Collateral Sciences.*

EDITED BY J. ADAMS ALLEN, M.D., LL.D.; AND WALTER HAY, M.D.

VOL. XXXI. — JANUARY, 1874. — NO. 1.

### Original Communications.

ARTICLE I.—*On a Marsh Plant from the Mississippi River Ague Bottoms, supposed to be kindred to the Gemiasma of Salisbury; with a Consideration of its Genetic Relations to Malarial Diseases.* Read before the Chicago Society of Physicians and Surgeons, November 10, 1873. By JOHN BARTLETT, M.D., Chicago.

*Gentlemen:* I invite your attention this evening to a plant from an ague bottom of the Mississippi river, with the purpose of considering the probability of its giving origin to the influence called malaria.

The theory of the origin of miasmatic diseases in the emanations of plants is not new. I shall not here recount its history, but will at once make reference to the most recent exposition of the idea as set forth in the remarkable paper of Dr. J. H. Salisbury, of Cleveland, Ohio, published in 1866, in the *American Journal of the Medical Sciences.*

The main facts in this paper may be thus stated: In the secretions of a large number of patients affected with malarious disease, and resident upon ague levels, Dr. Salisbury discovered, as the only extraneous bodies constantly found, minute oblong cells. These cells were recognized in the saliva, perspiration and urine of every patient examined. The next step in the investigation was the discovery of similar cells arising from the malarial soil. Upon glasses placed at night over its surface, which was in this case a partially desiccated and peaty prairie bog, Dr. Salisbury found in the morning these same bodies. Growing upon the ground over which his glasses had been placed, were plants which he regarded as of a palmelloid type. In a number of instances he was enabled to point out, in a striking manner, the association of these plants with localized attacks of ague; and in several cases, in which, for the purpose of experiment, sods of ague soil had been left in sleeping apartments, at a distance from malarious regions, he found that ague was developed in the previously healthy persons who were thus caused to be exposed to the emanations of the marsh earth. In every inhabited locality, where Dr. Salisbury found these plants growing, intermittent or remittent fevers, or both, prevailed, in proportion to the extent and profusion of the palmellæ.

In regard to the pathology of the disease, Dr. Salisbury says: "The lesions of intermittent fever are confined mostly to epithelial structures, showing, quite conclusively, that the exciting cause acts primarily upon the parent epithelial cells. . . . . . These derangements consist in the altering and enlarging of glandular structures, and in inflammations and alterations in structure and function of the mucous, epidermic and serous surfaces. All other abnormal manifestations are either symptomatic of these, or are the result of previous disease in the organism. All the glands in the body belong strictly to epithelial tissue, and are made up mostly of parent epithelial cells. These structures are affected in time and extent apparently in proportion to their importance in either organizing and assimilating products for nutrition, or disorganizing them for elimination. . . . . The exciting cause, inhaled, taken into the system with food and drink, and absorbed by the skin and mucous membranes, comes into direct contact with the epithelial cells, spread over and covering the entire body, both internally and externally, wherever there are any ways by which external bodies may enter the organism."

"Hence, the epithelial cells make up the first tissue of the system with which these poisonous bodies come into contact. They have to pass through these cells, before they can enter the systemic circulation, and reach the vascular tissues. In passing through these cells they derange them so as to poison the products therein organized. In this way the other tissues, including the ganglionic and cerebro-spinal systems, become involved. As the epithelial cells of the glands—especially those of the spleen, mesentery and liver—are the most largely engaged of any in organizing nutrient products for the other tissues, these glands are the most severely taxed, and are the first to suffer extensively from the poisonous palmellæ: hence it is that in these we so frequently find grave lesions. When the tissues have become poisoned to a certain extent, there is a reaction on the part of the system—an effort of nature to eliminate the poisonous products already in the body. This effort is the paroxysm which constitutes what we call the disease."

As regards the ague plant in the system, he says that the examination of the urine of several hundred cases of intermittent and remittent fever, "establishes the fact that ague plants, the same as grown upon the soil, are constantly developed in the system of patients affected with intermittent fever, and that the urinary organs constitute one important outlet for the elimination of this fever-vegetation. . . . . . The ague plants occur in the urine in the form of little cottony flocks, so small that they are scarcely noticeable by the naked eye, and too few in number to communicate turbidity to the excretion. They are very light in color, highly transparent, and appear to be developed in the bladder, pelves of the kidneys and ureters, often in considerable numbers."

In reference to the mode of elimination of the noxious element, Dr. Salisbury says: "The exciting cause must be carried out of the organism through those excretory channels (the urinary organs and respiratory apparatus) which nature has provided for the elimination of effete and abnormal products."

These views of Dr. Salisbury met with opposition. Dr. Horatio Wood, Professor of Botany in the University of Pennsylvania, published in the *American Journal of the Medical Sciences*, for 1868, a review of Salisbury's article, which perhaps did more to shake the faith of the profession in his discoveries than any similar publication. The objections urged by Dr. Wood were cogent, and forcibly put, and were well calculated to put a quietus upon Salisbury's claims. In 1871, becoming interested in the statements made by Prof. Salisbury, and being at that time in Keokuk, Iowa, near the great ague bottoms of the Mississippi river, I determined to find, if possible, the plant somewhat indefinitely described in his paper. Several examinations of the soil, within the limits of Keokuk, and in the rocky bed of the canal in course of construction there, were made without result. In September of that year, I determined to examine the slough margins in the lower river bottom opposite the city, and invited Dr. J. P. Safford to accompany me. Pointing out to him the low alluvial soil to be examined, and giving him as accurate an idea as possible of the plant described by Salisbury, I requested him to search for it, while I was engaged in visiting a patient.

Upon reaching the marsh, my friend presented me with a sod containing numerous plants, answering somewhat to the description given him. The specimens secured we compared subsequently with Salisbury's account, but we could only conclude that the plant found was a variety of the gemiasma, differing from them in several particulars; chiefly, perhaps, in respect to its size. Salisbury, in a letter, described the larger of his plants as capable of being seen by the aid of a powerful lens. These were as large as rape seeds, and therefore visible to the naked eye of a passing observer.

I was at that time unprovided with suitable means of investigation, and did little more than observe a correspondence between the prevalence of ague and the vigorous condition of these plants. Specimens of the growth were sent to distinguished botanists of this State, and to Mr. M. C. Cooke, of London, but no responses were received from them indicating that the plant had been previously described.

In August last, I determined to continue this investigation, and for that purpose visited Riverside. This town is situated on the Des Plaines river, twelve miles from Chicago. For several years past, ague has prevailed there very extensively. I sought for the plant on the margins of the river, confident of my ability to discover it. In this, however, I was disappointed; none could be found. Subsequently, in an interview with Dr. Fox, who has long practiced in the neighborhood, it was learned that the ague did not prevail there this year, but, on the contrary, there was a marked exemption from it. Lemont was next visited, a town twenty-six miles distant from Chicago, on the Illinois and Michigan canal. For several years it had suffered severely from malarial fevers. Dr. W. P. Peirce, a prominent practitioner of that place, informed me, upon my arrival, that there had been no prevalence of ague in that locality during this season, and that he knew of but two cases, and one of these was of foreign origin. With the assistance of Dr. Peirce, I proceeded to search for Safford's plant, in localities where ague had prevailed in previous years, and where, in Dr. Peirce's judgment and my own, the necessary conditions of soil would lead to the expectation of such a prevalence. No plants were found after an extended search. I then wrote to Dr. Safford, requesting him to visit the ague fields of East Keokuk, and forward to me some specimens of the plants discovered by him. In response, a meagre supply was received, with the statement that these had been procured with difficulty; that in the slough beds where we had found them growing in myraids in 1871, not a single plant had been discovered after hours of most careful search. The specimens forwarded had been found in another locality nearer the river. In 1871, in East Keokuk, the ague was epidemic. It might be said that every resident had the disease, and even those living on the adjacent bluffs, one hundred feet above the level of the river, were generally affected. Dr. Safford made the examination, referred to above, on the first of last September; he learned on inquiry that the ague had not prevailed in East Keokuk during the present season.

About the Des Moines rapids of the Mississippi, the Government is constructing a canal, seven miles in length, on the west side of the river. Since the commencement of this work, ague has at times prevailed among the laborers and their families, dwelling in shanties near the shore. The physician of that locality is Dr. G. F. Jenkins, of Keokuk. The ague prevailed especially along the canal in 1871–2. In the former year, Dr. Jenkins stated to me that he doled out to his patients thereabouts, an ounce of quinine daily. Whether the ague plant was growing at that time in that region, is not known. Dr. Safford and myself had found some specimens in the canal bed, in the lower part of the work.

On the fifteenth of September, Dr. Safford renewed his visit to

East Keokuk, and also requested Dr. Jenkins to examine the soil about the canal in his locality. On the extensive slough margins in East Keokuk, where no plant could be discovered two weeks before, they now existed in quantities exceeding the crop of 1871. *The ague was exceedingly prevalent.* Dr. Jenkins also reported that in the bottoms above Keokuk, where his practice was located, the plants were even more abundant than at East Keokuk, and that in the neighborhood, where two weeks before he had only two cases of ague, now "there was not a soul who was not affected with some form of malarial fever."

In July, 1872, I was called within a few days to five cases of ague in a small house near the corner of Clark and Division streets, about the centre of the burnt district on the North Side. This part of the city of Chicago, previous to the fire, was not liable to malarious affections. At the time mentioned, many of the lots were vacant, weeds covered squares of ground, and many cellars contained more or less water, either in consequence of defective drainage, or of leakage from adjoining hydrants. The house referred to above was in the rear of a blacksmith's shop in which some of the boarders were at work. Across Division street, to the south and a little to the east of this shop, at a distance of one hundred feet, was a pool of water in a cellar, produced by the leakage from an imperfectly closed hydrant. Of the five patients, the three first seized were laborers in the shop, and slept in the second story of the house, the windows of which were nearest the cellar referred to, and some one hundred and thirty feet distant from it. The other two patients worked at a distance from the house, and slept in a room in the rear of the one described. The disorder affecting those at work in the shop was obstinate; the attack of the two others was mild. The greenish mold on the margins of the collection of water described was examined at the time, but having no lens, I failed to recognize the plants. In September of this year I was called to the same house; two of the inmates were affected with ague. The soil of the cellar being examined, Safford's plant was found there. The fact was reported to the Board of Health, the hydrant was repaired and the pool partially filled in. One month afterward the ague patients relapsed, and upon examination the plant was found still flourishing on one of the sides of the former pool.

DESCRIPTION OF PLANT.

Safford's plant consists of body and what would appear to be a root. The body, or globe, consists of a wall enclosing a cavity. The layers of this wall are two; an internal, structureless envelope of a dull white color, like the retina in the cadaver, and an outer green wall, apparently resting upon the first as a basement membrane, which is much more complicated. It is composed of a great number of green cells; these are circular, and enclose green contents. The contained material seems to be divided by lines running across the cell, which do not, however, display any definite arrangement. At this point of development the cells furnish the observer no indication as to the granular or cellular condition of their contents. When injured they appear to discharge other very small and greenish cells of a simpler construction. The green wall cells do not adhere very tenaciously to the white membrane. They are readily detached from the latter by gentle friction and maceration, and float off on to the root or other adjacent body. Of the construction of the cavity of the plant within the white membrane I have no knowledge. It seems to be a simple sac. The globe of the plant, at maturity, collapses, the upper circumference falling in upon the lower in such manner as to leave to the view a cup, in place of a sphere. At first glance it would seem that the upper hemisphere of the globe had been thrown off, and that the observer was looking into the concavity of the lower hemisphere. More careful examination will show that the globe has collapsed, its contents escaping, and the upper half of its wall falling down upon the lower. The collapsed plant generally presents the cell wall unbroken. Occasionally the upper depressed half is slit open through its centre; frequently, along the margins of the cup, at the junction of the depressed and stationary portions, there are lacerations of the wall. When the soil containing the plant is removed from its natural bed and placed in different conditions, the collapse of the globe seems to be precipitated. The walls, examined immediately after having fallen in, appear of a darker color, as if moistened. The cavity of the plant contains a colorless fluid, which, it is presumed, is spontaneously evacuated when the globe collapses. It is forcibly ejected if the plant be punctured. I have never had an opportunity to examine it with a higher power than 200; I can therefore say nothing of its composition; it is

PLATE I.

*Representing an " ague field" : for degrees of magnification, see measurements below.*

### REFERENCES TO PLATE I.

A—A group of mother plants.
B—Group of collapsed plants.
C—Collapsed plants showing acicular crystals, bearing globules.
D—Group of crowded collapsed plants, developed in depth.
E E—Groups of crystalloid bodies.
F—Granular crystalloid body.
G—Green vine.
H H—Crystalline thread continuous with vine, bearing various prominences, etc. [plants.
I—Crystalline threads bearing green cells with cellular walls, supposed to be young mother
K K—Pieces of crystalline thread, variously colored—black, blue and carmine.
L—Crystalloid body putting forth green cells, supposed to be young mother plants.

### REFERENCES TO PLATE II.

A—Plant, showing globe and "root."
B—Collapsed plant; a forest of acicular crystals growing on its borders. [few).
C—Specimen of the blood of the writer, containing germinal atoms (blood discs relatively too
D—Acicular crystal, with globule putting forth contents. [beyond the visual distance.)
E—Spore of Safford's plant. (Cut too pronounced; a good representation when held just
F—Same, by polarized light—shaded portion, ruby red; light portion, green. [from it.
G—Spore; two germinal atoms near it; representation as of colony of atoms putting forth
H—Wall cells, as seen washed from the mother plant.
I—Imaginary section of collapsed plant, showing development of wall cells into spore cases.

Measurements.—Diameter of largest plant observed, 1-14 of an inch; its root, near the globe, 1-40 of an inch; wall cells, 1-1300 of an inch; crystalline thread, neither the largest nor smallest, 1-1000 to 1-7000 of an inch; germinal atoms, less than 1-15000 of an inch. No opportunity was had to take measurements of the spores and globules. These are about the same size, and, it is supposed, about 1-2000 of an inch in diameter.

PLATE II.

*For degrees of magnification, see measurements on opposite page.*

probably simply nutritive. Under certain conditions, as when an attempt is made to preserve the plant in glycerine, the green wall, losing entirely its cellular character, becomes rumpled up, and massed upon the inner tunic.

The root, or what seems to serve as such, is, in length, about six times the diameter of the plant. The trunk of the root soon puts forth a number of branches which seem to terminate in points, the latter becoming bulbous when soaked, as in glycerine. The root is white and translucent; not smooth, but having an appearance as if the surface were covered with granules. It is hollow, the fluid sometimes seen within giving the shaft the appearance of a glass tube containing water. It seems continuous with, and similar in structure to, the white wall membrane; or rather, this membrane appears to be an expansion of the root material. Dr. Safford regards the cavity of the root and body as continuous; he thinks he has seen the green cells of the wall within the hollow of the root. I have never observed such an appearance. The green cells of the globe wall were often seen floating upon, under and about the root, and massing together in its branches. In such specimens, however, it has been easy to recognize the fact that these cells have been washed from the body of the plant—the spot on the globe from which they have been removed being readily detected by the bald appearance of the denuded white membrane.

The plant varies greatly in size; perhaps the average diameter of the mature globe would measure $\frac{1}{20}$ of an inch. They are, of course, occasionally so small as not to be detected without a lens; the largest specimens measure $\frac{1}{14}$ of an inch. The cellular character of the green wall may be detected by a good lens, this coat appearing granular under such a power.

At the time of my reception of the samples of the soil from Keokuk, I was of the opinion that the collapse of the globe, the falling in of its upper circumference upon its lower, was the final act of vitality on the part of the plant. I thought that its spores existed in the cavity of the globe, and were thrown off to produce a new plant, in the act of collapse. It was, however, soon apparent that a collapsed globe did not cease to grow. The cells of its walls were observed enlarging, projecting from the basement surface, elongating, and in this manner becoming distinct from their fellows. The green coating of the mother plant is almost invariably covered with a white crystalline substance, which is abundantly scattered over the soil also, and everything in the vicinity of the plant. This white crystalline material was found so invariably and intimately associated with the plant, that it seemed highly probable some connection existed between them. Salisbury called the substance referred to, an aggregation of spores, and declared that he had seen with a high power the individual spores composing the mass. At the time of commencing my recent investigations, I was under the impression that this substance was adventitious—that it was, in fact, a species of dust. Upon placing the plant under the microscope, it was discovered that, after collapse, it underwent the following additional phases of development:

While the distinctive growth of the wall cells is in progress, the cup of the plant, made up by their aggregation, becomes deepened, so as to form a deeper and more cylindrical cavity, which, when a number of collapsed plants are crowded together, gives the mass a honey-combed appearance, the color being a light and brilliant green. The margins of the cells of this honey-comb (the margins of the collapsed globes) become coated with the white crystalline matter, of a duller and more bluish tint than that described. From this project innumerable short, acicular, vitreoid stems, each of which is surmounted with a brilliant globule, as of hollow glass. Meanwhile, two striking growths may be noticed near the plant and crossing over it,—one, a green vine, of a color similar to that of the cells; the other, a crystalline thread, resembling a crystal of the muriate of ammonia. Of these the green vine is the larger, and much less frequently seen; the crystalline thread is abundant, crossing and recrossing the plant, projecting into the air and over neighboring crevices. This growth, which appears to resemble the thread of ordinary mycelium, but which is different from it in various important particulars, is essentially the same as the green vine described above. The vine has been seen denuding itself of its green envelope and exposing the crystalline threads beneath. These filaments are not invariably white; they may be pink, are rarely blue, and at times they have an iron-mould, or even dead-black, hue. The larger specimens are as thick as fine sewing thread; their structure appears to be fibrous, and they are jointed, the joints bearing green-colored buds, also of a fibrous texture (apparent when they are viewed by transmitted light), and having a crystalloid appearance when examined by reflected light. Sometimes the thread has strung upon it, as it were, a disc of white crystalline matter, convex and comparatively smooth on one surface, and rough and jagged on the other. Occasionally the thread bears a stem surmounted by a small cone resembling a pine-apple, and still more rarely, it supports a candelabrum of branches, each branch surmounted by a series of minute globules of crystalline matter. Rarely the acicular crystals originate from the thread. The crystalline thread, when recent, is motile; extremities of it projecting upward into the air, or overhanging a cliff, may be seen to sway to and fro, as a grain stalk, in the breeze. A specimen of it, a quarter of an inch in length, placed in glycerine between glasses, writhed like a snake; and a ribbon of this substance which formed in six hours in a specimen of semen from an ague patient, exhibited undulatory movement, and occasionally rotated on its axis.

By a power of two or three hundred diameters, numerous short pieces of very small white or black crystalline filaments may be seen lying upon, and about the old collapsed plants. The green vine is tortuous; its surface is very adhesive; small worms coming in contact with it are unable to release themselves, and quickly perish. The crystalloid bodies, when viewed upon the plant, present the appearance of a fine crystalline white powder, resembling quinine, or small crystals, as of common salt. On the ground they are larger. Some specimens are quite large—as large as rape seeds. Some have a rounded outline like that of a pebble, or door knob; others have a broken appearance. Some appear to have a fibrous, others a hyaline structure. Occasionally the crystalloid bodies are of a reddish-brown color, or of a rusty hue, often they are of a glistening black tint, like that of cannel coal. All these varying shades are sometimes noted on one piece. The darker crystalloid specimens resemble more nearly an assemblage of atoms than those which are white. When viewed by polarized light these bodies appear to be marked, in a direction perpendicular to that of the fibrous structure (when this is apparent), by a series of dark, finely-scolloped lines. From the crystalloid substances also, seem occasionally to proceed the various projections, prominences, etc., described as belonging to the crystalline threads. The developed wall cells of the collapsed globe disappearing in the process of their complete development, nothing remains of the mother plant but a saucer-like pit in a slatecolored ground. The crystalline bodies and filaments have a longer life.

On concluding these general observations, I proceeded to inquire particularly into the connection between these growths; and with that view, a portion of a vigorously growing collapsed plant bearing acicular crystals, was seized and viewed under a low power. The wall cells then appeared to have become elongated, bottle or pestle-shaped, and in consequence of violence, exhibited patulous orifices, from which, in some instances, the contents were escaping. These contents were cells not distinguishable, except by their smaller size, from the wall cells of the uncollapsed globe. The slide was left in this state for a few hours. On my return, the parent wall cells were found collapsed, and the contained cells were not to be seen. The slide appeared to have been moistened, and was completely crowded with bodies, which under the power employed—seven hundred diameters—seemed to be minute non-nucleated cells. In attempting to estimate their size, I observed that new and smaller cells were constantly coming into view. It was concluded, therefore, that a protoplasmic fluid was under examination, out of which constantly originated the minute cells just described. The fluid was probably produced by the cells turned out of the cellular elements of the globe wall left on the slide. These cells emitted by the wall cells will be termed "spores," and the minute particles originating in the protoplasm, will be designated "germinal atoms." These latter had already exhibited, on the slide, a formative instinct. A double row of them stretched across the glass, one and another row appeared within these, and finally, before the observer was aware of it, a crystalline thread had formed. This was not at first round—it was such as had been seen on a "palmella" field—tape-like and twisted upon itself, appaıently, at about every half inch, as seen under a magnifying power of fifty diameters. Another row of cells soon ranged on either side of this filament, and formed two heavy brownish-colored lines, between which and the crystalline thread minute particles appeared to be developing. These latter were, one might say, points of verdification. Lastly, the green colored envelope of the crystalline thread was formed by the coalescence of these points with the brown line. Meantime, other particles were aggregated into white crystalline masses, or into bodies resembling granular casts from the kidneys. This and the self-multiplication of the cells was the limit of activity of the formative atoms.

Here was an interesting discovery: particles resembling those described by Salisbury as "spores," and found by him in human secretions, and upon slides suspended over ague plants, were recognized as extending from, and associated very intimately with, a vegetable growth, if not identical with, at least somewhat similar to, the plant described by him. And yet these particles were not spores—they were multiplying germinal atoms, which were seen to produce, *not the mother plant*, but crystalloid substances and crystalline threads intimately associated with it. I had seen the Salisbury spores (germinal particles) before, when crushing a matured globe upon the slide, but their extreme minuteness rendered their identification difficult. They had also been observed on slides suspended over plants. Whenever an object supposed to be a spore came within the field of the instrument, it had been my custom to cover it first with glycerine, and then with a delicate piece of glass. These attempts to prepare bodies suspected to be spores had been frequent, but in each case futile, as there resulted in the preparation not spores but wreaths or whorls of very minute cells. I was therefore well prepared, on recognizing them to be germinal atoms, for the following theory as to the plant's history:

The collapsed globe is the completion of one phase of the plant's life. From its cells are produced spores containing a protoplasmic fluid, which begets atoms, that in turn give birth to the crystalline thread and crystalloid bodies—these constituting the second phase of the existence of the fungus.

Leaving now, temporarily, this recognition of the probable character of the Salisbury "spore," I proceeded to investigate still another phase of the plant's development. Amid the disappearing collapsed globes, I observed proceeding from the crystalloid substances, and the crystalline threads, dark green cells with cellular walls, which were supposed to be young mother plants. After frequent and careful examinations, it seemed to me probable that the crystalloid bodies begat the mother plant; were in fact the seed of the ague fungus, fertilized, perhaps, by pollen in the globules of the acicular crystals. From these globules I had seen a colony of minute particles extruded, which appeared to remain inactive, that is, manifested no power of multiplication or formation. I have seen the white crystalloid bodies disintegrate, crumble down into particles, and have observed them covered with small, green, cellular plants, which seemed to originate from their surface. That part of the crystalloid mass not occupied by the plants resembled a piece of moistened white sugar, and presented here and there greenish spots, apparently the points of origin of other growths, similar to those more fully developed on other parts of the substance. The walls of the green cells originating from the crystalloid masses, and growing from the crystalline filaments, under my observation have never attained full size, for want, it is presumed, of proper conditions of growth. As they enlarge, their color changes from a light to a very dark green.

It will doubtless occur to some of you, that in this description several plants have been confounded in one. It is quite possible that such is the case. It is apparent, however, that the real ague plant, if such be included in this description, is that which begets the multiplying cell here termed germinal atom. The crystalloid substances and mycelium-like thread, are regarded as phases in the development of Safford's plant, because I have obtained from this, both in its globular and collapsed stages, germinal particles, which have given rise to these crystalline bodies and filaments; on the other hand, these bodies, in turn, give origin to plants resembling the young of the growth here described as the mother plant.

By placing slides close over the malarial soil at night, I was enabled to secure the spores. Upon passing a slide so exposed under a power of one hundred diameters, occasionally there would appear in the field a dark spot apparently about the size of the shaft of a pin, peculiar in nothing except its circular outline. When more highly magnified, this spot was found to have something of the appearance of the wall cell. They are generally perfectly circular, presenting the appearance of having an outer wall, a narrow intermediate light space and a denser centre. The ring described as a light space generally appears to be occupied with a row of dimly discernable cells. This appearance of minute particles in circular rows is occasionally recognizable also in the outer circumference of the dense centre. Toward the middle of the spore, this appearance is lost, the dark color being broken only by several irregular lines passing over its field. By polarized light under a power of 1000 diameters, the spore seemed divided into three spaces, as if by the drawing within its circle of two curved lines, the convexities of which approached one another near the centre of the spore. The central space, resembling the letter *x*, was of a ruby red color; the lateral spaces were green. These are the spores described as disappearing in glycerine. Left upon a slide, they may persist long without change. Sometimes a single germinal atom may be seen resting on the margin of the spore; at other times a shoal of particles may be noted proceeding from some point in its circumference. Free germinal atoms, and the glass-like globules which have been called pollen cells, are also occasionally found upon the slides. The stem of these globules appears to extend beyond them, or rather, from the surface of the cell at a point opposite to the attachment of the stem, a very short rod projects parallel with, but not in, the axis of the stem; it is a little to one side of the central line of the globule. Occasionally both stem and rod exist on specimens caught on slides. More commonly neither of these appendages are found upon such globules. Upon the suspended slides, quite a mass of the collapsed plant, containing a number of spores, has occasionally been found; sometimes the crystalline thread seems to be thrown upon the glass.

The germinal atom is the simplest form of a cell; when viewed with a power of 1500 diameters no nucleus or cell wall is to be observed. These particles appear as a slightly oval disc, with the palest blue or yellow tint. They strongly refract light, and by this property they may sometimes be distinguished from other particles, as specs of dust upon the eye-piece, or minute globules of glycerine, which they very much resemble. By screening the light from the field, they become conspicuously but not brilliantly lucent. The atoms vary in appearance in different secretions. In the saliva and urine they appear as in the protoplasmic fluid of the plant. In the blood they are circular, and have a yellow tint. When numerous upon a slide, they seem to overlie one another in part, so as to present an imbricated appearance. When a slide is densely crowded with the particles, it has an appearance as if frosted, or covered with wet snow. An atom taken from the tongue after having remained on the slide one hour, measured $\frac{1}{15000}$ part of an inch. At first to be seen with a high power only, they may be, after a growth of a few hours, advantageously studied with a power of 200 diameters. In the blood of two ague patients, yellowish cells, having a diameter one-fifth that of the blood corpuscles, were observed. In one specimen which had been allowed to stand uncovered for twenty-four hours, the cells had disappeared, but crystalloid bodies were present, and crystalline threads had pierced through the thickest mass of blood. Like results followed examinations of my own blood. From my tongue and buccal membrane were obtained spores and germinal particles; these latter grew and formed their peculiar product. Upon examining my urine at different times, germinal atoms have been found to appear five minutes after voiding, to increase to a large number in half an hour, and to form quickly quantities of crystalline bodies and filaments. Crystals forming in the urine, as those of the oxalate of lime, seemed to serve as nuclei of deposit; they speedily became covered with what by transmitted light seemed to be a greenish coating. This, however, by reflected light was found to resemble the usual crystalloid bodies. In one instance a collection of crystals of the oxalate which were grouped well together were covered with a continuous mass of substance appearing by transmitted light like a yellowish brown mold. In two specimens after the urine had stood (uncovered) on the slide for twenty-four or thirty-six hours, an elongated pear-shaped green cell was found growing, apparently from a crystalline body. This cell was peculiar in shape, and in having two lines or folds running laterally across it; it resembled a spore case of the *puccinia graminis*, and corresponded exactly with certain forms of cells intermingled with wall cells in a specimen of Safford's plant in my collection. Crystalline filaments were voided from the bladder, at least they were detected on a clean slide in half a minute after passing the urine.

This report would be incomplete were I to omit to state whether the occupation of my blood by these germinal particles had an influence upon the system. These plants have been growing in a very imperfect way (for it seems impossible to imitate their native habitat) outside of my window for about six weeks past. I have examined the whole field, one foot square, often during the day, and pieces of the soil, as large as a microscopic slide, have been under observation for hours together. For a month past, the particles described have been observed in my blood; at the present writing they are abundant. In fields of the microscope, with three hundred and twenty-five diameters, from twelve to fifty particles have been counted. For two weeks past I have had decided symptoms of remittent fever. Occasionally quinine has been taken, and always with the effect of diminishing, or removing the threatening symptoms.

Upon receiving plants from Keokuk, specimens were given to the accomplished botanists of this city, Profs. Babcock and Munroe, with the request that they should classify them. Meanwhile the above description was written, detailing simply what had been observed, botanical technicalities being purposely avoided. The report of these gentlemen upon the plant is now at hand. It is, they say, the Hygrogastrum of Rabenhorst, or the Botrydium of the Micrographic Dictionary. Upon reading the description of these growths furnished, the classification made by the botanists seemed to me quite correct, so far as the mother plant is concerned. But the authorities referred to make no mention of the development of the collapsed plant, or of the crystalloid substances and filaments To my inquiry of the botanists, whether it was probable that the mother plant should undergo the phases here described, they unhesitatingly declared that such a development was contrary to all analogy; and that it was highly probable, that the green vine, the crystalloid bodies and crystalline thread, with the knobbed acicular crystals and germinal particles, belonged to a distinct growth, probably to a fungus, parasite upon the Hygrogastrum.

### LOCALITY OF GROWTH.

In regard to the localities where the plant is generally to be found, I can only offer my own limited experiences. At East Keokuk, on the east bank of the Mississippi river, there is a series of small islands. These are separated from the main shore and from one another by narrow channels called sloughs. The borders of these islands are low, and at low water project outward as shelving beaches of sand. During the fall and spring the entire area of these lands is often overflowed, the water, upon its subsidence, depositing in those places where the current is slowest a greater or less amount of alluvial matter. The plane of the bottom varies to such an extent that with every day's decline of the waters there is exposed a large surface of moist alluvium. The ponds in the more elevated places disappear as the drying process continues, and new ones of lower level become cut off from the channel.

Some of the various shutes through the islands are, in every dry season, converted into a line of stagnant pools. It was on the margins of these that we found the plant described. The soil was porous and humid; at a distance of two rods from the water's edge the feet of the pedestrian left an impress, yet with care one could approach quite near the water without soiling the uppers of the shoes. The soil had a dull greenish appearance, from the deposit upon its surface of the green scum (*spirogyra*) left by the receding water. The ground was fissured in all directions, the crevices in the soil being sometimes several inches in depth, and an inch or more in width. In them, near the pond margins, the water stood within a few inches of the surface. Frogs, muscles, snails and insects were numerous. The soil was clothed with but little vegetation; it was bare of grass, but a very fine and short green moss was abundant. There was another species of moss which, for want of a better name, we called "stellate." It consisted of a number of small, thick, oblong leaves proceeding like radii from a common centre, and lying flatwise upon the ground, forming thus a disc from half an inch to an inch or more in diameter, with a plicated surface and crenated margin. The fine moss was assumed to be evidence that the soil was favorable for the growth of the plant, and the stellate patches were found to be still more intimately associated with it. The discovery of the stellate moss was quite sure to be followed by the finding of the ague plant.

The plants grew most plentifully on that belt of soil which lay between the very moist margins and the outer line of soil too dry for their growth. They were sometimes abundant on the margins of the fissures, and they were frequently found growing on the sides of the crevices, several inches below the plane of the surface. The plant is not confined to the moist, marshy soil described. It has been found growing in moist spots among the grasses at the roots of trees, and on the islands described at some distance from, and at some elevation above, the pond level.*

* It is difficult to cultivate these plants. I have not successfully done so. If not freely watered, the surface of the sod dries in the sun and wind; if the earth be made too wet, the plants do not flourish. The plan by which I succeeded best was to keep the sods in the shade, in a box with six or eight inches of garden earth under them. They should be moistened often, not by pouring water over the surface, but, by means of tubes conveying the water to the bottom of the box. Perhaps the plant might be more successfully cultivated in

OBJECTIONS CONSIDERED.

Reference has been made to the article of Dr. Horatio Wood. I desire to notice his objections and arguments against Salisbury's theory, with a purpose of inquiring how far they affect the view of the origin of the malaria given in this paper. As Dr. Wood's objections seem to embody all that could be said against Salisbury's theory, I desire to state them all, though it will be plain that a number of them would not have been urged against the theory here set forth.

Dr. Wood states that solution of quinine did not kill palmellæ; on the contrary, they flourished in it.

There are various points in the history of palmellæ which make it almost impossible that they constitute malaria; they do not grow in the dark; they could not, therefore, be supposed to flourish in the body. Frost lays a heavy hand on malaria; it does not kill palmellæ; on the contrary, they seem to flourish in an icicle. Prof. Leidy slept with various species of palmellæ, without disease ensuing. Dr. Wood has lived with palmellæ, and swallowed them by the thousand.

These are certainly very strong objections, as urged against the palmellæ with which Drs. Leidy and Wood experimented; but were these the plant to which Dr. Salisbury referred as the cause of ague? Dr. Wood candidly states that he does not know that they are; saying, with a just reflection on the exceedingly unsatisfactory description vouchsafed by Dr. Salisbury, "Prof. S.'s descriptions of his genera and species are so vague and destitute of character, that it is impossible to settle the question of identity, or to make any approach thereto."

a glass case arranged and managed like a so-called "fernery." In order to study the ague soil to advantage, it is desirable to put some of it in a box made of perforated tin, of the size of a microscopic slide, and about three-quarters of an inch in depth. It should be provided with a sort of tester of wire and tin, for holding a slide, when it is desired to secure spores; the frame work should be removable. The box may be placed upon the stage of the microscope, a piece of cloth protecting the instrument. When not under examination, it should rest in a cavity accurately cut for its accommodation, in a large sod of malarial earth, in order to prevent, as far as possible, drying of the soil, and to allow of its being properly watered. A good power in looking over the field is a two-thirds objective, giving a magnification of from seventy to two hundred diameters. The one-and-a-half inch objective may often be used to advantage, and occasionally a higher power, as a one-fifth, will be desirable.

It will be seen that none of these objections necessarily apply to Safford's plant as a causative agent of malaria—it is not a palmella. While quinine may not kill palmellæ, it may yet arrest the multiplication of the germinal particle here described. Palmellæ may not grow in the dark, but I have observed the germinal atom of Safford's plant multiply vigorously when deprived of light. The ingestion of palmellæ may be harmless, while the introduction into the blood of a rapidly multiplying cell may be hurtful.

Dr. Wood asks, "how could ague be cured, but by a long continued exhibition of some remedy capable of exerting a poisonous influence on palmellæ?" "There are," he continues, "diseases caused by fungi in the blood, but their history differs from that of intermittents" (referring to their producing a local and not a systemic effect). Again: the period of fruiting of algæ does not correspond to the prevalence of intermittents. Vegetable decomposition is an acknowledged necessity for the generation of malaria; palmellæ are independent of this. "The experience of the British army in Wallachia" (Walcheren?), says Dr. Wood, "is enough to set at rest the whole question of the genetic relation of malaria and algæ. The troops were encamped on a plain whose surface was composed of sand, so dry that no vegetation could exist upon it but a few heath plants."

The action of quinine in arresting the multiplication of cells in the lower order of plants out of the body, offers a ready explanation of its curative agency in malarious diseases, upon the theory of the cause here put forth.

There would seem to be nothing in the clinical history of malarious diseases contravening the theory of their production by multiplying cells, as suggested; judging from analogy, the effects of the entrance of such atoms into the system would be both general and local.

As regards the period of fruiting of Safford's plant, I cannot speak positively. I have never looked for it earlier than the beginning of September. It was then in full development, and as it speedily completes its cycle of growth, I know not why it may not be assumed that the infecting spores are being sent forth from the ripening of the first plant, early in the season, till the arrest of their growth by the frosts or floods of the fall.

Dr. Wood, in stating that vegetable decomposition is an acknowledged necessity for the generation of malaria, is probably in error. An opinion which is pointedly denied by such authors as Wm. Ferguson, John Bell, Flint and Aitken, can hardly be said to be acknowledged.

In regard to the question of the disappearance of the cause of ague here assigned after the fall frosts, all the facts known to me are as follows: In the latter part of October, Dr. Safford wrote that the plants in East Keokuk were disorganized by rain-storms and cold; that they were in a gelatinous condition, unfit for examination. More recently, Nov. 4, he wrote that, after careful examination of the field, he could not discover a solitary mother plant. There were numbers of the collapsed globes, but in these, the wall cells were shriveled and shrunken. Dr. Safford sent me some specimens. My examination substantially confirmed his observations; I found on the sods received but one or two wall cells in a perfect state. I observed, however, that the crystal-line threads were still growing, and that upon them were a few healthy looking cells which I have described as young plants. Dr. Safford states his conviction that, for the entire arrest of the growth of the plant, it will be found that a degree of cold sufficient to freeze the ground to the depth of half an inch or more will be required.*

Dr. Wood states that the experience of the British army, when encamped upon a dry, sandy plain, and assailed by malarious fevers, is sufficient to set at rest the whole question of the genetic

* Dr. Safford writes as follows: "I have made a last visit to the 'palmellæ' to-day (Nov. 3d). I have found them, as I expected, all dead or disorganized. I made thorough and careful search, and failed to find a single uncollapsed plant; cups there were in abundance, but these showed changes induced by storm and frost. I found them in what may be termed three stages of decay. 1st, greenish yellow cups, covering the ground like moss; 2d, cups of snow white appearance; 3d, beautiful carmine cusps, which gradually became a simple carmine incrustation upon the soil. I have seen all these changes going on in the same cup at the same time. I have long suspected that the reddish earth I have often found where I knew palmellæ had been, but had died out, was the remains of the plant; but I had never had demonstration of the fact till now." I found the Doctor's description accurate; the crimson collapsed plants were striking objects in the field sent. The cups described as greenish yellow were peculiar; they appeared to me to be the young plant, not collapsed, but *disrupted* by the weather; they were small, with cellular walls, and there was an appearance of one wall within the other, as in an opening bud.

relations of algæ and malaria. The question of the cause of malaria has been much obscured by the traditional reports of these army experiences, which have been transmitted unchallenged for generations. The following facts are quoted in many text books as showing an exception to the rule that malarial diseases originate in localities where there exist heat, moisture and appropriate soil; namely: Malarial diseases prevail on the heights of Gibraltar. They were rife among the English troops who, during the Spanish campaign of 1809, encamped upon the Guadiana, on the rocky heights of the confines of Portugal. Diseases referred to malaria prevailed among the British soldiers who, in 1794, were quartered at Rozendaal, in South Holland, upon a sandy plain, incapable of supporting any other vegetation than stunted heath plants. On the Alentejo land, situated upon the Tagus, opposite the city of Lisbon, where the soil is superficially dry, sandy and flat, residence exposes to malarial fevers. Finally, in the case cited by Dr. Wood, soldiers of Britain, when stationed upon a dry, sandy plain on the island of Walcheren, suffered unprecedented losses from miasmatic diseases.

Very naturally associating mountain heights and sandy plains with aridity and absence of vegetation, the student of malaria, accepting without question as isolated and independent facts, such statements as have just been cited, is lead to believe that malarial diseases may *originate*, as well as prevail, on a barren mountain or a desert waste. It is absolutely essential for the defenders of the theory of the origin of miasmatic diseases from vegetable germs, to show that such cases as those cited, form no exceptions to the general rule that conditions favorable to the growth of low forms of vegetation are essential to the production of malaria. I will, therefore, beg the indulgence of the Society, while I review the several statements referred to, with the view of showing that in no single one is there anything to prove that malarial disease originated under circumstances contravening the theory of vegetable origin.

Dr. Aitken says of Gibraltar: "On the summits of these rocks arise springs. The slightest frost produces fissures, into which fungi, *as mould*, and other vegetable matter, insinuate themselves. The rock of Gibraltar is known to be percolated with water, so that we can hardly conceive of a more pestilential focus of disease, when the causes necessary to the formation of fungi or miasm are considered."

Dr. William Ferguson, in a report on the sufferings of the British army in Spain, observes: "The retreat was made along the course of the Guadiana river, at a time when the country was so arid and dry for want of rain that the Guadiana itself, and all the smaller streams, had in fact *ceased to be such*, and were no more than lines of detached pools in the courses formerly occupied by the rivers. . . . In some of the hilly ravines that had been watercourses, several of the regiments took up their bivouac, for the sake of proximity to the stagnant water-pools that remained among the rocks."

I concur with Dr. James Johnson in the opinion, that no one familiar with the habitats of malaria, can find anything subversive of the ordinary theory of its origin in the occurrence of marsh fever among soldiers bivouaced *for the sake of convenience* "in the bed of a half-dried ravine and near stagnant pools." It was in just such pools, in the rocky bed of the Mississippi river, laid bare during the construction of the government canal at Keokuk, that I found Safford's plants growing.

Rozendaal, where the British suffered from malarial diseases on a sandy plain, in 1794, is located in Holland, on a bed of alluvium, in a malarious region, that is referred to in a geographical encyclopedia as "a country that draws fifty feet of water," and "is everywhere intersected with canals and ditches." The town of Rozendaal is situated on a watercourse, and it is encircled about ten miles to the west and to the north by another stream. It is but thirty miles west of the island of Walcheren, the topography, climate, etc., of which, presently to be given, may be supposed to be common to the two places. Without inquiry as to the condition of the troops upon reaching this plain, I call your attention to this extract from Dr. Ferguson's account: "On digging, it (the sandy soil) was universally found to be percolated with water to within a few inches of its surface, and this, far from being putrid, was perfectly *potable in all the wells of the camp*." We find here the soldiers using for camp purposes the surface water of a swampy region, vieing with India in its power of engendering diseases of a malarious character, aud we are asked to accept the case as one proving the power of a sandy plain to beget malaria!

In regard to the Alentejo land, opposite Lisbon, we discover that "the surrounding country is perfectly open, very low, and flooded with water during the whole of the rainy season. It is after the rainy season that the sickly season approaches." The proof of the non-vegetable origin of marsh miasm should hardly be sought for in the instance of the occurrence of ague in a low river bottom, completely overflowed for a period previous to the unhealthy season. On the dry, sandy shores of the Mississippi river, several feet above the water level, in depressed spots where the sand, though not wet, was moist, I have observed Safford's plant to flourish.

As bearing on the facts respecting the occurrence of disease on the sandy plain of Walcheren, I quote the following extracts: "Zealand is a region more completely enclosed by and sunk below the level of the water, than any other part of Holland. It consists of nine islands, formed and environed by branches of the Maise and the Scheldt, as, passing from the state of rivers into friths, they unite with the ocean. The mariner, in approaching, sees only the points of the spires peeping above the immense dikes thirty feet high which defend them from inundation. The soil is moist and rich. . . . The island of Walcheren is low, nine miles long and eight broad, and is subject to inundations. It has good arable and pasture lands. Flushing is the seaport, and Middleburgh is the capital."

As to the general sanitary condition of the island, I quote Mr. John Webb, the inspector of hospitals, writing when the ravages of disease had begun, Sept. 11, 1809: "Independent of existing records of the unhealthiness of Zealand, every feature of the country exhibited it in the most forcible manner; the lands communicating with the sea covered with the most noisome ooze; every ditch loaded with matter in a state of putrefaction; the whole island little better than a swamp; scarcely a place where water of a tolerable quality could be procured."

Now, what were the circumstances which preceded the fatal occupation of the sandy plain by the British? The army that besieged Flushing lay entrenched under its walls, "without other defense," says Dr. Johnson (who was on the spot), "from the sun, the rain and the dew, than some straw or brush-wood. Generally, indeed, the men had the humid earth for their beds, and the canopy of heaven for their curtains. When Flushing surrendered, a pause (fatal to military operations) ensued. A species of torpor, or rather, exhaustion, resulted, and then it was that the remote cause of fever, viz., vegeto-animal miasmata began to make its deleterious impression. But when we discovered that a boom had been stretched across the Scheldt, and that the surrounding country was inundated, and that various other insuperable obstacles interfered with the ulterior objects of the expedition, then, indeed, the depressing passions, and some other predisposing or exciting causes, communicated a fearful activity to marsh effluvium, which rivaled in its effects anything which has been seen in tropical climates. The French general, too, having opened the sluices, and *partially* inundated the country around Flushing, increased the force of the epidemic. Indeed, the road leading from the last mentioned place to Middleburgh, [nearly through the centre of the island?] might, at this time, vie, in respect to its insalubrity, with any through the pontine fens of Italy."

Another officer of the expedition wrote: "Toward morning we found ourselves wrapped in that chill, blue, marshy mist, rising from the ground, that no clothing can keep out, that actually seems to penetrate the inmost frame; the island was covered with a sheet of exhalation, blue, dense and fetid." Lord Chatham wrote, under date of Oct. 29: "The morning fogs began to be heavier and more penetrating, the soldiers were carried into close barracks at Middleburgh, where the fever raged more and more," etc. Dr. Johnson complains "that the army did not avail itself of some local advantages that presented themselves among those noxious islands," in that the soldiers were not tented on the elevated sand hills on the windward side of the island, "a site which would in all probability have kept them entirely out of the range of *those exhalations* which *covered* the country below." The island of Walcheren was notoriously unhealthy. In 1747 Sir John Pringle has recorded the experience upon it of English batallions, very similar to that of 1809.

Charles Knight, the historian, writes, referring to the contemplated expedition against Flushing, "every one who had thought or read knew what would be the consequence of sending 40,000 men to Zealand in August, and of their continuing there for two or three months." Napoleon wrote: "Before six weeks, of the 15,000 English on the isle of Walcheren, not 1,500 will be left."

It will be noted that the soldiery consisted of regiments, in which the marsh effluvium had already manifested "a fearful activity."

In considering the origin of malarial disease the important fact that it has a period of incubation should not be overlooked. This period, commonly, according to Dickson, seven days, not unfrequently extends over months. Were there reasons then to believe that the dry sandy plain of the Walcheren encampment was entirely free from miasmata, ample cause for the disease which prevailed could be logically sought after in the exposure of the army to the ordinary causes of malaria existing before Flushing. Indeed, Watson states that many of these very men who were exposed to miasmatic influences at Walcheren, did not experience its disastrous results until they had returned to, and resided some months in England.

On an alluvial island, of an area to be compassed with a radius of five miles, sunk below the sea level, recently half inundated, intersected with canals and ditches in the worst hygienic condition, with a climate notoriously malarious, its main road at that time as pestiferous as the pontine marshes, troops, enfeebled by warfare, despondency and extraordinary exposure on malarious shores, among whom miasmatic disease had already assumed a "fearful activity," are encamped, without tents, and without good water, on a sandy plain, at that season of the year when malarial influences are the most powerful. Marsh fevers make unprecedented havoc, and for several generations, medical writers have followed one another in citing the fact, as establishing the law, that malaria may originate on a dry sandy plain. And as late as 1868, the advocates of the theory of the vegetable origin of malaria are confronted with the statement, that the experience of the British army on the plain of Walcheren settles the question at issue, *against them!*

In the history of medicine it might be difficult to find an example in which so false a "fact" has for so long a time stood as a bar to the progress of truth. The advocates of fungal origin of paludal fevers may, at least, demand that observations bearing upon their theory be made anew, and with especial reference to the presence or absence in the infected localties of the agents assigned by them as the cause of disease.

Dr. Beale, in his work on Disease Germs, refers frequently to the production of systemic disease by vegetable germs, in such a manner as to lead the reader to infer that it was a matter of demonstration, that such an occurrence was an impossibility. His arguments are of no force as regards the plant and disorder under consideration. His chapter headed "Some difficulties which prevent us from accepting the Vegetable Germ Theory of Disease," is closed thus: "It may, therefore, be affirmed that the matter which forms the active virus or poisonous material (of contagious diseases) does not exhibit the properties of any vegetable or animal parasitic organism yet discovered and identified." This deduction is a complete summary of his argument; and I need not further occupy the time of the Society with reference to it, inasmuch as its bearing is upon a form of disease not here under discussion, and since the germinal atom here described is not included in the organisms referred to by Dr. Beale, as hitherto discovered and identified.

CORRESPONDENCE BETWEEN AGUE AND AGUE PLANTS AS TO LOCALITIES, ETC.

I now call your attention to the localities, conditions and circumstances in which malarial diseases arise, with the purpose of indicating a correspondence between these localities, conditions, etc., and those which pertain to the plant which we are considering. The following facts concerning the circumstances of origin, prevalence, etc., of paludal fevers, are taken from standard writers; and that the parallelism, which it is my purpose to point out, may be the more striking, I have written the laws of ague, and the corresponding known or highly probable facts regarding the plant, in adjacent columns.

First it will be noticed, that the localities in which malarial fevers abound, are those suitable for the growth of the plant described. "Agues have always been observed to be the diseases of moist or marshy districts, and to prevail most in low, swampy, and humid countries, where seasons of considerable heat occur. The vicinity of marshes, or of a district that has at some recent time been under water; the banks of great lakes, and the shores of great rivers and seas, where the water flows slowly, and in some places stagnates, in shallow rivers, over land alluvial, low and flat; extensive flat tracts of wood, where much moisture is constantly present, where the process of drying is uninterrupted, and yet the surface constantly exhaling humidity;—these are some of the terrestrial physical conditions in which the paludal and the litoral fevers are found to abound."

Malarial diseases are strictly localized in prevalence.

This is of necessity a fact in regard to marsh plants.

An average heat of at least 60 degrees for two months is necessary for the development of ague; its violence and mortality are greatest in tropical climates.

We have seen the ague plant develop in large quantities in two weeks. But it must be borne in mind that, in many places, it may be necessary that a long drying process shall have gone on in order to fit the soil for the growth of the plant. For both preparation of soil, and maturing of a sufficient number of plants, some months may be required.

Malaria most generally prevails after rains followed by great heat, by which the surface is gradually dried.

These are conditions manifestly necessary, in many districts, for a state of soil suitable to low forms of vegetation.

As a general rule, the nearer the plane of habitation approaches the level of the marsh, the more violent is the ague poison.

This law is palpable as regards any theory involving emanations from marshes as causative of disease.

Sometimes ague prevails more on an eminence near a marsh, than on a level with it.

The explanation of such facts may find an illustration in an occurrence detailed by Dr. Salisbury. After the capture of Nashville, barracks were erected on a high eminence, selected as a matter of sanitary foresight. This hill, which had been noted for its salubrity, was soon found to be more prolific of malarial fevers than the plain below. Upon its summit, breast-works had been erected, thus causing the exposure of an extended surface of fresh, moist earth as a bed for the growth of ague plants. This soil was, in fact, found by Dr. Salisbury to be covered with such growths. In this case, spores wafted from the ague fields below, found soil perhaps even more congenial, at the very door of the habitations of the troops stationed on the hill.

The draining of dams and ponds, and the first culture of new soil often induce ague. It may be developed in previously healthy places by turning up the earth, as in making excavations for foundations, railroads and canals.

By draining, wet soil is placed for a time under the most favorable conditions for the growth of the plant. And upturned soils may catch the spores of the fungus not sufficiently abundant in the air to beget disease, and grow and multiply them enormously. The same may be said regarding the excavation of earth for canals, etc.

In proportion as countries are cleared up and settled, periodical fevers disappear.

The cause of this change is conceded to be drainage, whereby marshes, etc., in the vicinity of habitations become unfit for the vigorous growth of the plant.

Malarial poison has an affinity for dense foliage, which seems to have the power of accumulating it, when lying in the course of winds blowing from malarious localities.

The fact of foliage arresting and accumulating malaria will not be surprising upon the theory here set forth. Slides of glass, without any preparation of their surfaces, are found to arrest and suspend the spores and germinal atoms. It is manifest that the leaves of trees would be much more competent for such a purpose. We are told, however, that pine trees, which present a much less perfect barrier to particles in the air than trees of denser foliage, are equally effectual in arresting malaria. Prof. Dickson expresses the opinion that the pine tree is antidotal to marsh poison, citing the fact that ozone is found in the atmosphere contained in a bottle half filled with turpentine and exposed to light and air. Beale states that a minute trace of some of the ingredients of tar dissolved in water, is destructive to many of the lower forms of life. To an antagonism of this kind may also be referred the statement regarding the freedom from malaria about which grow the Jussieua Grandifolia or the Eucalyptus Globulus.

Not all marshes are malarious.

This fact may readily be accounted for, inasmuch as without the ague plant there may be no ague. As at

East Keokuk, prior to September first, the marshes were there as usual, but no fever appeared till a short time afterward, when the plants were abundant.

Ague exists where there are no marshes.

The ague plant may grow in comparatively dry places, provided that a degree of moisture is constant. It may be assumed, however, that this law is deduced by authors from the often quoted Walcheren experience, a review of which has already occupied your attention.

Periodical fevers never prevail in the thickly built portions of cities.

In such localities, there are few spots offering conditions suitable for the growth of the plant.

Covering the soil of ague districts with water arrests the disease. A violent rain storm diminishes the amount of miasm.

This fact was remarked by me when the Mississippi river overflowed the ague fields. The plants covered with water, or even soaked by rain, are in a jelly-like condition, incapable, in all probability, of emitting spores.

The local prevalence of ague in the autumn is checked by a decided frost.

The growth of Safford's plant arrested by frost, is, in all probability, entirely checked by cold sufficient to freeze the ground for half an inch.

NATURE OF MALARIAL DISEASES.

The nature of malarial diseases, if the theory involved in this paper be correct, is plain. The germinal atoms, less than one-fifth of the diameter of the blood corpuscle, readily enter the blood with the air inspired, or with the food or drink. When there, they may induce disease by a catalytic action, described by Liebig as an influence of contagion, by which the mere presence of a third inactive body invites, induces, changes between other substances hitherto resting in equilibrium with one another. Catalytic action is now however, by many chemists, set aside as an unnecessary theory regarding results to be explained upon principles in accordance with direct laws of cause and effect.

According, then, to the more recent theories, the action of the foreign germinal atom in the blood may be supposed to be that of a ferment, and the malarial diseases to be literally zymoses. The atoms produce changes in the blood, as the atom of the yeast plant produces changes in a saccharine solution, by seizing upon elements of the sugar, and applying them to its own uses; meanwhile, in turn, depositing in the solution substances which may bear to the cell the relation of effete matters. The living germinal atoms feed upon the fluids of the blood, and induce farther changes in it by making it the depository of their excreta. The germinal atom of Safford's plant may induce changes in the blood as the very similar atom of the yeast plant, vinegar plant, etc., induces changes in fluid in which they are placed. Liebig was of the opinion that oxygen was essential to the process of fermentation, and other chemists have maintained that the process was one dependent upon ferments spontaneously originating in the fermentible fluid. Pasteur, however, by his masterly study of the subject, has shown that oxygen is not necessary for this process; nor can it originate of itself. The essential causative element in fermentation in all cases is the minute self-multiplying cell of a plant.

There are those who have opposed the theory of fermentation in the blood, regarding the process as one quite impossible in a vital fluid, and in fact, looking upon it as an occurrence only to be met with in the brew-house. Even Beale, the great advocate for the causative influence of germinal atoms in regard to cattle plague, gonorrhœa, vaccine disease, small pox, etc., is entirely incredulous of the fermentation theory. According to my idea, which I will say has been gathered from the study of Beale's writings on bioplasm, etc., fermentation is a process most likely to be taken on by the blood; in fact, I regard the transformations continually effected in the system by secreting cells and bioplastic atoms as strikingly analogous to the changes induced in fermentible liquids by the cells of plants. In both instances we have a compound fluid, and a living cell capable of producing transformations in it. Flint, when considering the mechanism of secretion, says: "There are certain anatomical elements (epithelial cells) in the glands which have the power of selecting the proper materials from the blood, and causing them to undergo catalytic transformation (into secretions)." Borrowing the phraseology of this physiologist, I might say: In ague there are certain elements (vegetable cells) in the circulation, which have the power of selecting normal materials from the blood, and causing them to undergo catalytic transformation into products abnormal, and noxious thereto.

It is to be assumed that the vital fluid is not disposed to take on the malarial fermentation, that upon the entrance of ague atoms into the blood, they are eliminated, and it is only when the production of atoms is in a certain degree in excess of the elimination that a paroxysm is induced. One atom, or a thousand atoms, may not excite the disease in those whose blood is indisposed to that particular form of change induced by the ague cell, or in a system in which the germs are rapidly excreted. We may, however, suppose that the quantity of atoms becoming excessive, either from an increase in the number entering the system, or from a failure of the excretories to cast out the usual quantity, a paroxysm results. For the full development of fermentative processes, a certain interval of time is necessary; we may, therefore, expect a period of incubation in malarial diseases. This period may be a few hours, or it may be months. In the latter case, it is supposable that some intercurrent condition of the vital fluids has rendered blood hitherto indisposed to undergo malarial fermentation, susceptible to it.

Lest some should deem this theory too bold, and void of foundation, I beg leave to call your attention to certain facts developed by Pasteur. In general terms it may be stated, that he found that certain fluids, capable of all forms of fermentation, preferred certain forms, though if pains were taken, any particular fermentation might be induced in them. Thus, the fluid *a* prefers to undergo the *x* fermentation, and if fermentation cells *x* and *y* and *z* are all placed in like number in the fluid *a*, it invariably undergoes the *x* fermentation; should you, however, interfere with this preference so far as to add a great number of *z* atoms, the *z* fermentation will result, or the *y*, as you may elect; other fermentible fluids, as *b* and *c*, prefer the *y* and *z* fermentation; meanwhile, while a fluid is undergoing any fermentation preferred by it, or forced upon it, any other fermenting cells present are dormant.

This has a parallel in clinical history. It is a familiar fact that while one zymotic disease is in action, as the scarlet fever, vaccinia may be held in abeyance, afterward to assume action. And a yet more striking fact is this—using the language of our theory—a patient may be undergoing the intermittent or remittent fever fermentation, the yellow fever atom enters his system, and at once, as it were, assumes control, the earlier form of fermentation ceases, and that of yellow fever proceeds.

Thus in a healthy system, the blood prefers to undergo the changes natural to it, preferring to submit, as it were, to the action of its own bioplasms and cells. A limited number of germinal ague atoms then have no influence, but if the system be crowded with them, the healthy processes cease, and the foreign cells hold sway.

An attempt to explain the phenomena of periodicity of malarial diseases has been made by the advocates of its fungal origin; thus, certain forms of spores exist in a given infusion, they gradually diminish, and finally subside. After an interval they reappear in incredible numbers, soon to disappear and to appear again as before.

According to these views, ague is, like the yellow fever and cholera, a portable and communicable disease. It is manifest that enough crystalline substance might be carried under the finger nail to infect the Mississippi bottom with ague. There are not wanting physicians whose experience has led them to believe that ague is communicable—perhaps their opinion is not without foundation. It should be borne in mind that ague poison, like the cholera virus, would depend for its propagation upon the contingency of the falling of its seed on soil suitable for its growth.*

According to Dr. Beale, the most conspicuous pathological result following the multiplication of germ cells in the system, is a blocking up of the capillaries by their aggregation in these terminal vessels, and perhaps by fibrine effused because of their presence. In cattle plague, a disease produced by just such a cell as I have described as an ague atom, this blocking up or closing of the capillaries was the primary pathological ĕvent. Following it was an increase of development of the bioplasts of the part, transudation of the cattle plague cell through the walls of the capillaries, and great development of it in the connective tissue. I see no reason why the pathological changes resulting from intermittent and remittent fever

* In this connection I will refer to a suspicion which observation has aroused in my mind, that cholera is sometimes produced by the use as food, of certain large plants, as cabbages. May it not be that these plants, often growing on soil competent to nourish fungi, accumulate the cholera poison on their leaves, and thus are the means by which disease germs enter the system?

might not be referable to like actions set up in the capillaries of the liver, spleen, etc., by the development of the ague atom.

The corroboration which the zymotic theory of malarial diseases derives from a consideration of its *juvantia*, I will only refer to. Arsenic, quinine, the sulphites. etc., are well known to be destructive of the life of the lower order of plants. Their power over the disease may be ascribed to the destruction of the ague germ, or to the arrest of its development.

Before closing this reference to the possible action of the ague atom in the system, I wish to call your attention to a reference made by Dr. Wood, in his article against the fungal origin of disease. After maintaining that in whatever instances fungi were known to affect the human system, their effect was local — that no systemic disease was known to be produced by them, he proceeds as follows: "The nearest approach to the production of systemic disease by fungi. is seen in the affections of certain of the lower animals. Their spores have been found in the blood in some of these cases. . . . . They appear to act on the blood, as they do upon other tissues, producing a local disease of it, so to speak, giving origin to a steadily progressive train of symptoms. They feed upon the nutritive fluid, form filaments in it which pierce the walls of the vessels, and ramify through all the tissues. The most carefully studied of these affections is that which attacks the ordinary house fly. The first appearance of this disease is the presence of very minute oval cells in the circulating fluid, which cells increase in number, enlarge, grow into filaments, pierce the blood vessels, and ramify through all the tissues, gradually destroying them. In eight or ten hours after death, the filaments continue to grow, pierce through the surface of the body, and interlace over it, to form a whitish winding sheet." Dr. Wood regards it as doubtful whether in this instance the fungus was the cause of death ; it perhaps simply preyed upon a mortally stricken fly.

I regard this as a case in which a vegetable cell, endowed, like the ague atom, with the power of multiplication and formation, entered the blood and induced death by general and local disease.

### YELLOW FEVER AND CHOLERA DUE TO GERMINAL ATOMS (?)

There are two diseases which in the laws of their occurrence are so similar to malarial fevers, that it may be assumed that if the one be found to depend upon a fungus growth for its origin, a similar cause will be discovered for the other two. These diseases are yellow fever and cholera. In regard to the former, the actual facts tending to show its dependence upon a vegetable germ for its cause, are very meagre ; but they are nevertheless worthy of mention. Prof. Leidy, in 1854, among the matters detected in black vomit, mentions "crystalline bodies." Dr. Riddell discovered minute filiform algæ. Dr. Hassall detected ramose branches of the sporules of a fungus; branched and moniliform threads of a fungus. In 1867, Schmidt observed in black vomit granules with very dark and thick outlines, with a very clear and refractive centre, and fuller grown spores of fungi — the smallest germ having a diameter of one twenty thousandth part of an inch. From the spores filamentous tubes were developed. Any or all of these bodies may have been developed in the fluid from germs received into it after its discharge, but in the light of the facts now presented in regard to fungal developments in ague secretions, they present a certain interest, and perhaps some importance.

To such a plant as here described, might be referred many of the phenomena of cholera. We have only to suppose a cholera fungus capable of growth at certain temperatures, and under certain conditions, in order to explain the non-contagion, portability and communicability of this disease, as well as certain peculiarities in its clinical history. These peculiarities are, a period of incubation, and a marked difference in its modes of invasion. It may succeed an attack of diarrhœa, or may at once prostrate a patient and destroy life, in the manner of the agencies which produce congestive fever.

There is a fact in connection with its causation, to which I would here call attention. Niemeyer and others confidently affirm that the main source of communication of the disease, is to be found in privies. And it is a generally accepted statement that during the prevalence of the disorder, privy odors should be regarded as evidences of danger. In the "American Journal of the Medical Sciences" for 1868, Dr. Edgar Holden, in an article on the causation of attacks of intermittent fever on shipboard by a species of mold, known as "Thallophyte," says: "I am convinced that there is a way in which it [sulphuretted hydrogen gas] is responsible for certain diseases."

In the course of the article, he puts forth the opinion that the mold alone, and the hydro-sulphuric acid alone, are incapable of producing intermittent fever, but that in some way their association is competent to bring about such a result. It may be that sulphuretted hydrogen gas in swamps, and in the filthy localities of large cities, is essential to the perfect development of the ague and cholera fungus. But there is a much more important idea to which I desire to call your attention. In 1838, Boehm stated that in cases of cholera the entire intestinal tract teemed with a vegetation of micro-fungi; that innumerable round and oval, or more elongated corpuscles were there to be discovered. In 1848, Dr. Parkes recognized dark-yellow, or black, granules in the blood of cholera patients. In 1866, Prof. E. Hallier, of Jena, described spore-cysts to be found in cholera excreta—yellow or brownish-red bodies, consisting of a pale membrane enclosing highly refractive, colored spores. These spores are to be resolved into "colonies"—very small cells, called by their discoverer "micrococci." By cultivating these spores, Hallier succeeded in causing to grow from them a long, pale filament, from which spore-producing processes branched off. Subsequent observation led Hallier to the conclusion that the fungus, which, in its development, assumed five different forms, was, in fact, an urocystis. Analogous fungi occurring in the tissue of certain grasses, connected these discoveries with the idea of the earlier English practitioners in India, that the cholera was in some way connected with the rice plant. Hallier succeeded in developing in the tissues of such plants, watered with cholera secretions, a fungus which was apparently similar to that found in the cholera discharges. So strong was the expectation, aroused by these facts, that the cause of cholera had been discovered by Hallier, that two physicians, well qualified for the task, were detailed by the English government to investigate the subject. After a most carefully conducted research they reported as follows:

"1. No cysts exist in cholera stools which are not found under other conditions.

"2. Cysts, or sporangia of fungi, are very rarely found under any circumstances in alvine discharges.

"3. No special fungus has been developed in cholera stools, the fungus described by Hallier being certainly not confined to such stools."

Dr. McNamara also asserts his conviction that fungi peculiar to cholera discharges have not been discovered: that in them, as in other nitrogenous substances undergoing decomposition, fungoid growths occur. He positively affirms that no such growth can be detected in *fresh* cholera stools—the more recent the specimen examined, the more certainly will the absence of fungi be established.

After these declarations of most competent observers, Dr. Aitken says: "So far, then, as fungi are concerned in the spread of cholera, I am satisfied that we have no grounds for such belief."

It will be observed that there are certain elements of Hallier's fungus analogous to characters of Safford's plant. It has several remarkable phases of development, and presents filaments, spore-cases (here called wall cells), spores, and *contained atoms.*

Last summer, when the pathology of cholera was under discussion, and cholera stools were under examination, before this Society, I took occasion to make the following remark:

"The cholera paroxysm is regarded by many as an effort on the part of nature to rid the system of a poison, and with that idea in mind, the cholera ejecta are examined for the purpose of discovering the noxious element. If the phenomena of cholera result from an eliminative effort, that effort is generally unsuccessful. In view of this fact, would it not be more rational to look into the system, and especially the vascular system, for the cause of the disease?"

You will have anticipated my purpose in this reference. Hallier's idea, and that of his followers, as well as of the critical investigators of his position, was fixed solely, I believe, upon the presence of an *intestinal* fungus as a cause of cholera. The report of the British Commission, so far as can be discovered from extracts here accessible, has no bearing on the genetic relation to cholera of fungal elements *in the blood.* May it not yet be found that a multiplying cell of Hallier's plant (his micrococcus?) is the cause of cholera? The products of such a cell, the fungal character sought for in the intestines by the English investigators, might be as harmless there as the crystalline thread of Safford's plant in the bladder, and yet its multiplication in the blood might prove as fatal to the organism as pernicious fever.

The following considerations regarding the pathology of cholera will not be without interest in this connection. Nearly all pathologists recognize in the clinical history of the disorder, obstruction to the flow of blood in the capillaries—some referring this condition to spasm, others to paralysis of the vaso-motor nerves. Dr. Beale has invariably found obstruction of the capillaries to be the most palpable condition of disease, resulting from the multiplication of disease germs in the blood; and this obstruction has been detected by him in cholera cases. It should be here stated, that this accomplished microscopist incidentally notices the presence of minute germs of fungi in cholera blood.

AGUE FROM OTHER PLANTS—HYGIENE—DIAGNOSIS, ETC.

There is reason to believe that the phenomena of ague, or at least, paroxysms similar to those of intermittent fever, may be induced by emanations from several forms of fungus. Thus, Holden, in 1866, reports eight cases of intermittent on shipboard very directly traceable to the exposure of the patients to a species of mold known as Thallophyte which had rapidly and abundantly developed in the ship's storeroom, as Dr. Holden believed, under the stimulus of sulphuretted hydrogen gas arising from the bilge. And, according to Dr. Aitken, epidemics of malaria have been traced by Reid, in the Mauritius, and by Massey, in Ceylon, to very minute fungi of rapid growth, as a cause.

In the event of the plants described being the cause of ague, what will be the value of the discovery as a matter of hygiene? Localities where they prevail may be avoided, as in pitching a camp or founding a settlement. When the surface occupied by them is small, they might be destroyed, according to Salisbury's suggestion, by covering them with lime, ashes, or straw. If their territory of occupation is extensive, it might be necessary to change the condition of the soil by drainage, in order to secure their destruction. It is possible that some vegetation may be discovered to be hostile to their growth, as are reported to be the plant and the tree previously mentioned in this connection. Finally, by experimentation, some medicine harmless to the system may be found, which, taken continuously, would destroy them in the system, or at least arrest their increase in some such manner as quinine may be supposed to do. Where exposure is inevitable, the experiments of Pasteur would suggest the use of the cotton-wool respirator.

In the event of these theories being correct, we have in the microscopical examination of the secretions of the patient an excellent means of diagnosis, and having at hand the exciting cause, experiments made directly upon it would, without doubt, lead to great advances in the therapeutics of malarial diseases.

SALISBURY'S DISCOVERIES.

Before concluding, I wish to make some remarks regarding the discovery of Prof. Salisbury. Are his plants and Safford's the same? There is a want of correspondence between the description given by Salisbury of the gemiasma, and what I have called the "mother plant." On some sods sent from East Keokuk was what appeared to the naked eye to be a green mold. Dr. Safford had sent this growth as specimens of young plants of the kind discovered by him. Upon examination, the cell wall of the small globes was found to be cellular, and they were accordingly regarded as differing from the wall cells of the collapsed plant, which they resembled, and as being Safford's plant dwarfed by unfavorable change of situation. I have observed also that the cells growing from crystalloid bodies, and promising to become mature mother plants, soon ceased to grow in my ague field. They remained diminutive, and seemed to multiply by vegetative increase, one apparently giving origin directly to another. Now these cells correspond in appearance and size quite exactly to Salisbury's description of palmellæ. It may have been Safford's plant in this form of growth which he saw.

Dr. Salisbury describes the "spores" thought by him to produce ague, as "minute oblong cells, either single or aggregated, consisting of a distinct nucleus, surrounded by a smooth cell wall, with a highly clear, apparently empty space between the outside cell wall and nucleus." This description does not apply to the simple germinal atom described by me. It applies rather to the body which I have represented as a spore, excepting that this object, which is larger than a blood disc, could not be called a minute cell, "the most minute of all known organic cells." Unable to reconcile these differences, I am still disposed to believe that Salisbury's spore and the "germinal atom" may be the same body. I have observed that the atom appeared, when out of focus in one direction, as a granule; in the contrary direction, as an enlarged ring. Were Dr. Salisbury less expert as a microscopist, it might be supposed that he had deduced the idea of a nucleus and cell wall from such appearances — a deduction, it may be remarked, much more likely to be made in 1866 than at the present time. Multiplying and formative power is not assigned to the ague cell by Dr. S.

Prof. Salisbury refers to "salt-like incrustations," and speaks of them as disintegrating, and, if I rightly conceive his meaning, developing into palmellæ. He refers incidentally to "confervoid filaments," and to "green confervoid filaments," and, while he describes the plants as appearing in the urine, in a manner not observed by me, as "cottony flocks," and as "the same plant as grown upon the soil," he also states that he has found quite uniformly in the urine of ague patients, "the spores of a species of fungus—generally vegetating—*belonging to* the genus sphærotheca, and which is uniformly found growing on, and in, the larger species of palmellæ, the gemiasma protuberans." Also, that "in some cases of ague of long standing, yeast plants, *species* of Penicillium and Aspergillus, are also found, developing in large numbers, the mycelia often rising to the surface a short time after the urine is voided, producing fertile threads and fruit."

It is evident that I have worked in the same field with Dr. Salisbury. Further investigations will render what in the history of the "gemiasma" is now uncertain and indefinite, plain and clear; and, in the event of the cause of malaria being found associated with these plants, even in a manner far different from that suggested by Dr. Salisbury, to him will yet belong the credit of original and effective research in the direction which led on to the truth.

In closing this paper, I desire to state, as an explanation of the want of completeness of my study of the plant and its possible causative relation to malaria, that my investigations have been cut short by the disappearance of the fungus, and the diseases supposed to depend upon it. The results of my work thus far are put forth with the hope of securing such co-operation from other observers as may insure an early solution of the question at issue.

## Figures and Tables

**PLATE I. f1:**
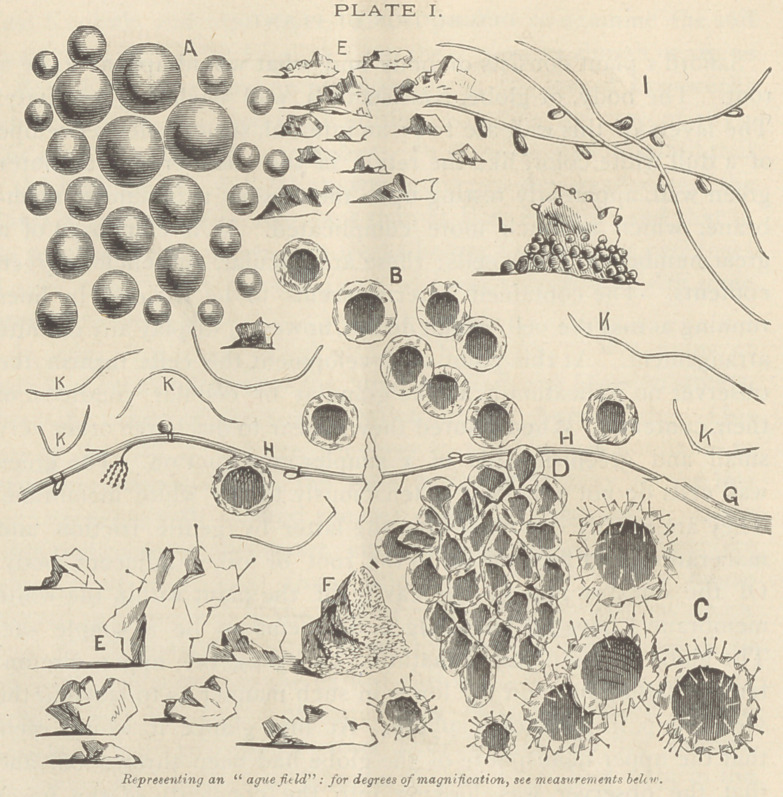


**PLATE II. f2:**